# Fusion of Infrared and Visible Images Using Fast Global Smoothing Decomposition and Target-Enhanced Parallel Gaussian Fuzzy Logic

**DOI:** 10.3390/s22010040

**Published:** 2021-12-22

**Authors:** Chaowei Duan, Changda Xing, Yiliu Liu, Zhisheng Wang

**Affiliations:** 1 College of Automation Engineering, Nanjing University of Aeronautics and Astronautics, Nanjing 211106, China; duanchaowei@nuaa.edu.cn (C.D.); xingchangda@nuaa.edu.cn (C.X.); lyl331982180@nuaa.edu.cn (Y.L.); 2 Shenzhen Research Institute, Nanjing University of Aeronautics and Astronautics, Shenzhen 518063, China

**Keywords:** fast multi-scale edge-preserving decomposition, targets/regions of source images, target-enhanced parallel Gaussian fuzzy logic, visual saliency map

## Abstract

As a powerful technique to merge complementary information of original images, infrared (IR) and visible image fusion approaches are widely used in surveillance, target detecting, tracking, and biological recognition, etc. In this paper, an efficient IR and visible image fusion method is proposed to simultaneously enhance the significant targets/regions in all source images and preserve rich background details in visible images. The multi-scale representation based on the fast global smoother is firstly used to decompose source images into the base and detail layers, aiming to extract the salient structure information and suppress the halos around the edges. Then, a target-enhanced parallel Gaussian fuzzy logic-based fusion rule is proposed to merge the base layers, which can avoid the brightness loss and highlight significant targets/regions. In addition, the visual saliency map-based fusion rule is designed to merge the detail layers with the purpose of obtaining rich details. Finally, the fused image is reconstructed. Extensive experiments are conducted on 21 image pairs and a Nato-camp sequence (32 image pairs) to verify the effectiveness and superiority of the proposed method. Compared with several state-of-the-art methods, experimental results demonstrate that the proposed method can achieve more competitive or superior performances according to both the visual results and objective evaluation.

## 1. Introduction

As is well known, infrared (IR) imaging is playing an increasingly significant role in various ground object identification cases, such as camouflage recognition and hidden targets [1]. IR images can reveal the thermal radiation difference of diverse objects, which can well distinguish the targets from their backgrounds. Due to the inherent property of being free from the influence of some bad conditions like smog, low illumination, etc., the IR imaging system has the strong ability to capture the targets well in all weather conditions day or night. However, IR images typically have inferior detail textures and low-definition backgrounds. Contrarily, the visible imaging technology is able to record the reflected lights of objects. The visible images can provide more considerable texture details and far greater clarity than IR images. Nevertheless, it tends to be affected by foul weather. To acquire sufficient information for accurate scenario analysis, users usually require to serially analyze multiple images with different imaging forms of a scene. No doubt analyzing multi-modality images of a scene, one by one, brings some problems (e.g., needing more time and more work) to users. To address the above problems, it is desirable to integrate multiple kinds of images into a single complementary and informative image [2]. The fused image can provide an enhanced vision of a scene and preserve the useful features of source images, i.e., the thermal radiation information in the IR images and the texture appearance in visible images [3].

The IR and visible image fusion has attracted a wide range of attention in the past few years [4]. The composite results of IR and visible images can provide more comprehensive information than any source images. An informative-fused image promotes the subsequent high-level processing of machine vision and facilitates scene understanding for humans. Due to the considerable merits mentioned above, IR and visible image fusion technology has been widely employed in various applications, such as military surveillance [2,5], concealed weapon [6], agricultural automation [1,5], pilot assistance [7], remote sensing [8], and biometric identification [9,10].

Despite quite a number of methods in the IR and visible image fusion field having achieved good results [1,2,4,5], many existing methods have several open-ended difficulties and suffer from some challenges [11]. Traditional multi-scale transform-based methods implement quite quickly and easily, but the fusion performances are poor in many conditions due to the luminance degradation and the loss of details [12,13]. Advanced learning-based methods often can have good visual effects, but the computational efficiency usually is not high [2,5]. To overcome the above challenges, effective image information extraction schemes and advanced image fusion rules are two directions to explore. Besides, the computational efficiency of IR and visible image fusion is an important issue in many practical applications. However, the requirements of practical applications are not often considered [1,11]. Therefore, the runtime efficiency should be taken into account as well.

**Figure 1:**
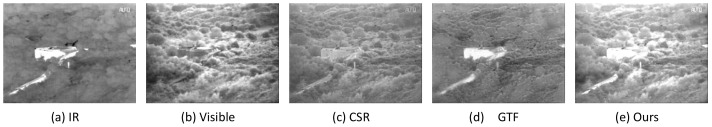
An example of the infrared (IR) and visible image fusion on ‘Bunker’ from the TNO dataset. From left to right: (**a**) IR image, (**b**) visible image, and (**c**) the fusion results by CSR [14], (**d**) GTF [15], and (**e**) our method, respectively.

In this paper, an IR and visible image fusion method is proposed based on the multi-scale decomposition using the fast global smoother, which aims to effectively extract the significant structural information of the source images. Two fusion rules (target-enhanced parallel Gaussian fuzzy logic- and visual saliency map-based fusion rules) are designed to highlight the targets and regions of interest both in IR and visible images, and also preserve rich details with high visual fidelity. Figure 1 shows the superiority of our presented method on the ‘Bunker’ example from the TNO dataset. It can be found that the IR image has outstanding targets, and the visible image contains abundant background information. Compared with the fusion results of CSR (convolutional sparse representation) [14] and GTF (gradient transfer fusion) [15], the proposed method can simultaneously fuse the thermal target information from the IR image and the texture information from the visible image well. In contrast, CSR suffers from brightness distortion, and GTF has difficultly preserving the detail textures in the visible image and the characters ‘AUTO’ in the top right of the IR image. The schematic diagram of the proposed image fusion framework will be illustrated at the end of Section 3.4.

We conduct extensive experiments to verify the effectiveness and superiority via comparing the proposed method with several state-of-the-art fusion approaches on 21 image pairs and a Nato-camp sequence (32 image pairs). Subjective and objective assessment results demonstrate the superiority of our method qualitatively and quantitatively. Moreover, the extension of the proposed method to multiple (more than two) source images is effective as well. The major contributions of this work are outlined as follows:

(i) An effective fusion method for IR and visible images is proposed using the fast global smoother to efficiently extract multi-scale structure information and well suppress the halos around the edges.
(ii) A target-enhanced parallel Gaussian fuzzy logic (TEPGFL)-based fusion rule is proposed to merge the base layers. The TEPGFL-based fusion rule can avoid brightness loss and highlight the significant targets in IR images and the high-brightness regions in the visible images. The fused results are more natural and consistent with the human visual system so that the fused results attract people’s attentions.
(iii) We present a visual saliency map (VSM)-based fusion rule using the Scharr gradient to merge the detail layers with the purpose of extracting rich details and textures. The Scharr gradient reflects the significant structure features of an image, such as edges, outlines, region boundaries, etc. The visual saliency map based on the Scharr gradient has the ability to enhance the detail textures and capture the significant structures of objects. Therefore, the proposed VSM-based fusion rule can obtain a fused image with rich details and high visual fidelity.
(iv) The proposed fusion method has high computational efficiency. The high computational efficiency facilitates the practical applications of the IR and visible image fusion.

The rest of this paper is arranged as follows. Section 2 provides the related works in the IR and visible fusion field. Section 3 details the proposed fusion method. In Section 4, experimental results and the corresponding discussion are presented at great length. Finally, Section 5 gives the conclusion and discussion on future work.

## 2. Related Works

Researchers in the image fusion community have developed various image fusion algorithms in the past few decades [1,2]. The representative fusion approaches can be roughly classified into the following categories: Multi-scale decomposition (MSD) [16,17,18], sparse representation (SR) [19,20], hybrid schemes [21,22,23,24], deep learning (DL) [25], and other novel methods [15,26].

MSD-based methods can be further categorized into two classes, i.e., multi-scale transform (MST)-based and edge-preserving filter (EPF)-based methods. MST-based methods include pyramid transform (PT) [27,28], wavelet transform (WT) [29,30], curvelet transform (CVT) [31], contourlet transform (CT) [32], non-subsampled contourlet transform (NSCT) [33], and non-subsampled shearlet transform (NSST) [34], etc. MST-based methods often use the popular “average” rule to fuse the low-frequency sub-band images while it employs the widely-used “maximum” selection or “absolute maximum” selection rule to merge the high-frequency sub-band images. MST-based approaches can fast achieve good performances in many cases. However, MST-based approaches suffer from serious artifacts, which limits the fused results [1,4]. In the past few years, the edge-preserving filter (EPF) has been introduced to the image fusion community [35]. Among the EPF-based methods, various filters such as the bilateral filter (BF) [23], rolling guidance filter (RGF) [24], guided filter (GF) [36], and cross bilateral filter (CBF) [37] have been used to extract the main structural information and preserve residual, smaller-scale details. Ma et al. [24] have used RGF to obtain the base and detail layers and utilized visual saliency map and weighted least square optimization to combine the base and detail layers. In [36], GF is used in the fusion rules to achieve good results in a manner consistent with human visual perception. Nevertheless, the fancy decomposition algorithms and elaborate fusion schemes based on EPF may consume significant computational power and time, i.e., the BF-based method [23]. The GF-based method [36] may bring about halo artifacts and contrast loss [38]. Due to using simple fusion rules, the CBF-based method [37] could suffer from blocking artifacts [22].

Recently, sparse representation (SR)-based schemes have shown to be remarkable methods [14,19]. Generally, the SR-based image fusion comprises four steps [2]. Firstly, source images are decomposed into the overlapping patches via a sliding window. Secondly, sparse coding is conducted on the vectorized patches to achieve the sparse representation coefficients using a learned over-complete dictionary. Thirdly, sparse representation coefficients are combined via the given fusion rules, i.e., weighted average and choose-max. Finally, the final result is obtained by the learned over-complete dictionary. Besides working as a decomposition tool, SR also can be used in fusion rules [19,20]. In [20], the low-frequency sub-band coefficients are fused using sparse representation. But SR-based schemes are usually time-consuming.

Furthermore, for the hybrid schemes [21,22,23,24] and other novel methods [15,26,39], the former combines the advantages of various algorithms while the latter adopts some uncommon but novel strategies. In [22], the simple mean filter has been used to perform a two-layer decomposition while the visual saliency detection using mean and median filters is obtained to construct the saliency and weight maps. In [15], an optimizing model-based method using gradient transfer and total variation minimization can highlight the targets in IR images well.

In recent years, state-of-the-art deep learning (DL) has been widely used for image processing due to its excellent feature extraction ability. Convolutional neural networks (CNN)-based methods have been proposed for the IR and visible image fusion in [3,25]. Recently, Bhalla et al. [3] have proposed a novel fusion method using fuzzy-based siamese CNN (FSCNN). Siamese CNN is used to extract salient features of source images for the first time. The fusion FSCNN-based algorithm has achieved excellent results on the fusion of IR and visible images. Besides, many other neural network models in the image fusion field have been widely studied [40,41,42,43]. In [40], Li et al., proposed a deep learning framework using imagenet-vgg-verydeep-19 for IR and visible image fusion. In [41], the deep learning framework with ResNet and zero-phase component analysis has achieved a good fusion performance. Raza et al. [42] presented a new fusion method based on the IR features and multi-scale dense network (IR-MSDNet) to preserve the important target features and key content. A salient target detection-based fusion network (STDFusionNet) has been proposed to merge IR and visible images [43]. Although DL has great potential for the improvement of IR and visible image fusion, DL-based fusion methods still have some open problems, such as lack of the ground-truth and large-scale trainable data for training images [44]. It is difficult to define a standard ground-truth for fused images [45].

## 3. Proposed Method

The presented fusion method based on multi-scale decomposition using the fast global smoother (MFGS) consists of three steps to conduct fusion on two pre-registered IR and visible images. Firstly, we decompose both the IR and visible images into a series of base and detail layer sub-images using MFGS proposed in Section 3.1, respectively. Secondly, the target-enhanced parallel Gaussian fuzzy logic (TEPGFL)-based fusion rule is adopted to merge the base layers with the goal of highlighting objects/regions in the IR/visible images and enhancing contrast of the fusion result. The visual saliency map (VSM)-based fusion rule using Scharr gradient is utilized to merge the detail layers with the purpose of achieving rich details and textures. Finally, the final fused result is reconstructed via combining the merged base layer and the merged detail layer. In what follows, we provide the entire fusion methodology in detail.

### 3.1. Multi-Scale Decomposition Using the Fast Global Smoother

A natural image with rich contents typically contains diverse structures with different scales and diverse objects with different sizes, which can provide humans with various information for scene understanding. To better describe a scenario, many applications in image processing and computer graphics often require the decomposition of an image into a piecewise smooth base layer and a set of detail layers. The base layers capture the main structural information, while the detail layers contain the residual smaller scale details in the images. With the goal of extracting the significant feature information of the IR and visible images fast and effectively, an edge-preserving smoothing method stemming from the weighted least squares formulation, called the fast global smoother (FGS) [46], is employed as the multi-scale decomposition tool in this work.

Similar but different to several popular filters including a bilateral filter (BF) or its improved version [23], weighted least squares (WLS) [35], and guided image filter (GF) [47], etc., the motivation to use FGS for decomposition is twofold. On the one hand, FGS has the preeminent artifact-free characteristics, as opposed to the common limitation of those fundamentally local filters [47,48] which have no ability to fully resolve the vagueness in regard to whether or not to smooth certain edges (often producing halos around edges) [46]. On the other hand, FGS solves a sequence of 1-D global optimization-based linear sub-systems other than the previous computationally expensive linear system solvers in the optimization-based methods, such as weighted least squares (WLS) [35] and L0 gradient minimization [49], etc. Compared with the edge-preserving filters such as the fast bilateral filter (FBF) in [23], rolling guidance filter (RGF) in [24], and cross bilateral filter (CBF) in [37], etc., FGS can more significantly accelerate FGS-based multi-scale decomposition, thereby the highly computational efficiency of fusion is achieved. The speed ability makes our proposed fusion method more suitable for practical application in future. The computational efficiency also needs to have considerable attentions, separately from good fusion performance [1,2].

#### 3.1.1. Fast Global Smoother

The FGS essentially utilizes a fast technique based on a highly efficient three-point Laplacian matrix algorithm designed over a d-dimensional spatial domain. Firstly, for a 1-D input signal $f_{x}^{h}$ and a 1-D guide signal $g_{x}^{h}$ along the *x* dimension (x=0,...,M) in a horizontal direction, the 1-D WLS energy function is given by:

(1) $$J\left(u^{h}\right)=\sum_{x}\left({\left(u_{x}^{h}-f_{x}^{h}\right)}^{2}+\lambda_{t}\sum_{i\in N_{h}\left(x\right)}\omega_{x,i}\left(g^{h}\right)\left(u_{x}^{h}-u_{i}^{h}\right)\right)$$

where $u_{x}^{h}$ denotes a desired output obtained by minimizing Equation (1), and $N_{h}\left(x\right)$ indicates a set of two neighbors for *x* (i.e., x−1 and x+1). $\lambda_{t}$ is the smoothing parameter controlling the balance between the two terms. Increasing the value of $\lambda_{t}$ can make the output $u^{h}$ more smooth. Generally, the spatially varying weighting function $\omega_{x,i}\left(g^{h}\right)$ is selected as a smoothness constraint with the rang parameter $\sigma_{c}$ to control the similarity between two 1-D signals $g_{x}^{h}$ and $g_{i}^{h}$. Mathematically, it is defined as follows:

(2) $$\omega_{x,i}\left(g^{h}\right)=exp(-∥g_{x}^{h}-g_{i}^{h}∥/\sigma_{c})$$

where, the 1-D guide signal ($g_{x}^{h}$) in Equation (2) being equal to the 1-D input signal ($f_{x}^{h}$).

Secondly, to compute the 1-D output solution $u^{h}$, we rewrite Equation (1) as a linear system using matrix notation as follows:

(3) $$\left(I_{h}+\lambda_{t}A_{h}\right)u_{h}=f_{h}$$

where the size of the identity matrix $I_{h}$ is M×M, and $u_{h}$ (or $f_{h}$) denotes the vector notations of $u^{h}$ (or $f^{h}$). $A_{h}$ represents the three-point Laplacian matrix with the same size as $I_{h}$. Then, Equation (3) can be expressed as:

(4) $$\left[\begin{array}{ccccc} b_{0} & c_{0} & 0 & \ldots & 0\\ \ddots & \ddots & \ddots & 0 & 0\\ 0 & a_{x} & b_{x} & c_{x} & 0\\ 0 & 0 & \ddots & \ddots & \ddots \\ 0 & \ldots & 0 & a_{M-1} & b_{M-1} \end{array}\right]\left[\begin{array}{c} u_{0}^{h}\\ \vdots \\ u_{x}^{h}\\ \vdots \\ u_{M-1}^{h} \end{array}\right]=\left[\begin{array}{c} f_{0}^{h}\\ \vdots \\ f_{x}^{h}\\ \vdots \\ f_{M-1}^{h} \end{array}\right]$$

where Equation (4) is a linear system with boundary conditions $a_{0}=0$ and $c_{M-1}=0$. Here, $a_{x}$, $b_{x}$, and $c_{x}$ indicate three nonzero elements in the *x*-th row of $\left(I_{h}+\lambda_{t}A_{h}\right)$, which can be presented as follows:

(5) $$\begin{array}{c} a_{x}=\lambda_{t}A_{h}\left(x,x-1\right)=-\lambda_{t}\omega_{x,x-1}\\ b_{x}=1+\lambda_{t}A_{h}\left(x,x\right)=1+\lambda_{t}\left(\omega_{x,x-1}+\omega_{x,x+1}\right)\\ c_{x}=\lambda_{t}A_{h}\left(x,x+1\right)=-\lambda_{t}\omega_{x,x+1}. \end{array}$$

From Equations (4) and (5), it is seen that $A_{h}$ is a three-point Laplacian matrix with nonzero elements only exiting in the diagonal, and the left and right diagonals, thereby solving $u^{h}$ becomes much easier.

The above-mentioned 1-D fast global smoother (FGS) algorithm can only deal with 1-D signals. For processing a 2-D image, similar to horizontal 1-D solvers, we perform the 1-D solvers again in a vertical direction. In addition, performing 2-D smoothing three iterations can obtain the final result with scarcely any streaking artifacts [46]. More importantly, it is faster (about 30×) than the WLS-based filter in [35].

#### 3.1.2. Multi-Scale Decomposition Using FGS

Inspired by scale-space theory in [50], we present a new multi-scale decomposition algorithm using FGS. Like pyramid-based decomposition schemes, our approach has a similar decomposition framework. Our algorithm has no up-sampling and down-sampling steps so that our algorithm can well suppress the information loss. Furthermore, using FGS instead of a Gaussian filter can restrain from the indiscriminate blurring, thus retaining such significant features as edges, outlines, etc. The FGS-based decomposition algorithm generally is comprised of four main steps. Mathematically, the procedure is depicted as follows.

*Step* *1:* For any one of *N* source images $I_{n}$ (n=1,...,N), $I_{bn}^{0}$=$I_{n}$ serves as the initial input image.
*Step* *2:* Make use of the fast global smoother to separate progressively larger structures of the input source image, meanwhile maintaining the edges.
  (6) $$I_{bn}^{l}=FGS\left(I_{bn}^{l-1},\sigma_{c}^{l},\lambda \right)\;\;l=1,...,L$$
  where FGS denotes FGS filtering, and $I_{bn}^{l}$ indicates the *l*-th base layer image. $\sigma_{c}^{l}$ is the rang parameter at the *l*-th level, and *l* is the multi-scale decomposition level currently. Here, $\lambda =\sum_{i}^{T}2\lambda_{t}$ (*T* is the total number of iterations. In our work, T is set to 3 suggested by [46]), and empirically λ=10. Additionally, $\sigma_{c}^{l}$ is also regarded as the scale control factor. When the structure scale in the image $I_{bn}^{l-1}$ is smaller than $\sigma_{c}^{l}$, the structure will be eliminated in $I_{bn}^{l-1}$ according to [50]. Let $\sigma_{c}^{l+1}=2\sigma_{c}^{l}$ in Equation (6) for extracting the progressively coarser structures.
*Step* *3:* The *l*-th level detail layer is obtained by:
  (7) $$I_{dn}^{l}=I_{bn}^{l-1}-I_{bn}^{l}\;\;l=1,...,L.$$
*Step* *4:* Iteratively executing Equations (6) and (7), *L* progressively blurry base layers and *L* gradually coarse detail layers can be achieved easily at different scales, respectively. With the FGS-based decomposition scheme, a source image can be decomposed into a collection of detail layers and a base layer as follows:
  (8) $$I_{n}=I_{bn}^{L}+\sum_{l=1}^{L}I_{dn}^{l}\;\;l=1,...,L.$$

**Figure 2:**
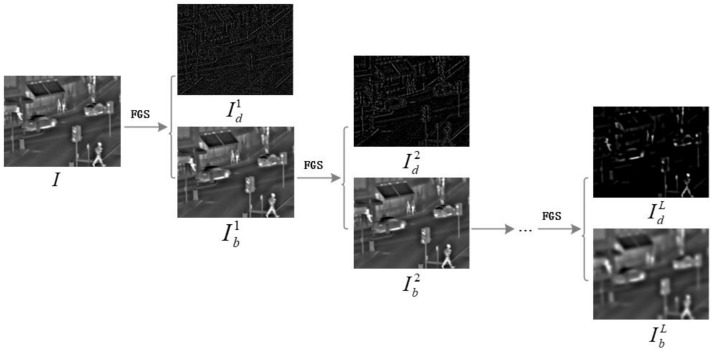
MFGS: Multi-level decomposition framework using the fast global smoother. *L* indicates the number of decomposition levels (L=9 tested on ‘Road’ here).

Using the FGS-based decomposition approach, a multi-scale decomposition scheme can be applied to each source image. At this point, a multi-level FGS-based framework is achieved, which is called MFGS. The MFGS framework is schematically illustrated in Figure 2. As can be seen in the top of Figure 2, the extracted scale structures of the detail layers become increasingly coarse with the growth of the decomposition level, which is consistent with the process of human visual characteristics.

### 3.2. Base Layer Fusion

As illustrated in Figure 2, the base layer $I_{b}^{L}$ in our decomposition framework typically contains the main low-frequency energy information, which displays the global contrast and overall appearance of a source image [22]. The fusion rule of base layers plays a crucial role in the visual performance of the fused image. The often-used “average” rule (AVG) is commonly selected to fuse the base layers or low-frequency sub-band coefficients in many cases for the reason that the AVG fusion rule realizes fusion in a simple and easy manner. However, along with simplicity of the AVG fusion rule suffers from a negative effect, that is, the contrast is obviously reduced. The contrast reduction makes the brightness of some objects/regions decline sharply. Fortunately, some researches have focused on this problem and achieved good performances in [15,24,51], etc. Nevertheless, Ma et al. [15] and Chen et al. [51] mainly focus on the targets in the infrared images, both of which ignore the bright regions in visible images, so that unnatural results that are not consistent with human visual perception are exported. Though the work in [24] considers the saliency features in both IR and visible images, the detailed texture unclarity still arises in the composite images. To address these issues mentioned above, we propose a target-enhanced parallel Gaussian fuzzy logic (TEPGFL)-based fusion rule inspired by [52] to merge the base layers. The major difference between TEPGFL and the fusion rule in [52] is that the latter adopts the single Gaussian fuzzy logic (SGFL)-based fusion rule to highlight the brightest targets/regions in IR and visible images but neglect other important targets/regions without the highest brightness. The fusion results could be better if the SGFL fusion rule in [52] had considered more the important targets/regions, which may be not the brightest targets/regions. The detailed discussion will be provided in Section 4.2. In this work, we propose a target-enhanced parallel Gaussian fuzzy logic (TEPGFL)-based fusion rule, which considers not only all significant targets in the IR image but also all important regions in the visible image. Furthermore, the target enhancement coefficient is designed to enhance objects/regions for improving the fusion performance. The TEPGFL-based fusion rule is detailed as follows.

Firstly, we select the Gaussian membership function to determine the degree of membership between the target and background for each pixel (*i*, *j*) in the IR base layer $I_{b1}^{L}$. Here, for a source image $I_{n}$ (n∈(1,2,...,N)), $I_{1}$ denotes the IR image and $I_{2}$ means the visible image.

(9) $$w_{b1}\left(i,j\right)=exp\left[-\frac{{\left(I_{b1}^{L}\left(i,j\right)-\mu_{1}\right)}^{2}}{2{\sigma_{1}}^{2}}\right]$$

where $w_{b1}$ is the fusion weight coefficient for $I_{b1}^{L}$. $\sigma_{1}$ and $\mu_{1}$ are the standard deviation and mean value of the IR base layer $I_{b1}^{L}$, respectively.

Then, the first initial merged base layer $B_{f1}$ is obtained by:

(10) $$B_{f1}(i,j)=w_{b1}\left(i,j\right)I_{b1}^{L}\left(i,j\right)+\left(1-w_{b1}\left(i,j\right)\right)I_{b2}^{L}\left(i,j\right).$$

Secondly, similar to Equations (9) and (10), we can compute the second initial merged base layer $B_{f2}$.

(11) $$w_{b2}\left(i,j\right)=exp\left[-\frac{{\left(I_{b2}^{L}\left(i,j\right)-\mu_{2}\right)}^{2}}{2{\sigma_{2}}^{2}}\right]$$

where $w_{b2}$ is the fusion weight coefficient for the visible base layer $I_{b2}^{L}$. $\sigma_{2}$ and $\mu_{2}$ are the standard deviation and mean value of the visible base layer $I_{b2}^{L}$, respectively:

(12) $$B_{f2}(i,j)=w_{b2}\left(i,j\right)I_{b2}^{L}\left(i,j\right)+\left(1-w_{b2}\left(i,j\right)\right)I_{b1}^{L}\left(i,j\right).$$

Finally, the final merged base layer $B_{f}$ is given as follows:

(13) $$B_{f}=C_{1}B_{f1}\left(i,j\right)+C_{2}B_{f2}\left(i,j\right)$$

where $C_{1}$ and $C_{2}$ denote the contrast enhancement coefficient for targets/regions. To reduce parameter complexity, we set $C_{1}$ = $C_{2}$ = $C_{bf}$. Parameter selection and analysis of $C_{bf}$ will be provided in Section 4.1.4.

### 3.3. Detail Layer Fusion

It is well-known that “maximum” (MAX) selection and “absolute maximum” (ABS-MAX) selection fusion strategies are the two most popular fusion rules broadly used in various fusion methods. However, they have certain shortcomings, such as ignoring detailed structures and introducing halo artifacts in the procedure of combining the detail layers [4]. Indeed, the detailed structures in the detail layers can expose the significant edges and major contours of the objects well. To acquire rich textures and details, a visual saliency map (VSM)-based fusion rule using the Scharr gradient algorithm is proposed for merging detail layers. Scharr gradient magnitude is often used in image quality assessment to measure the image quality due to its powerful ability of enhancing the outlines of objects [53,54]. In this work, Scharr gradient magnitude (SGM) is used as an activity level measure to extract the salient structures from the outline structure aspect. SGM can reflect the significant structure features, such as edges, outlines, region boundaries, etc. First, the SGM of a image *I* is defined as:

(14) $$SGM_{x}\left(i,j\right)=\frac{1}{16}\left[\begin{array}{ccc} 3 & 0 & -3\\ 10 & 0 & -10\\ 3 & 0 & -3 \end{array}\right]*I\left(i,j\right)$$

(15) $$SGM_{y}\left(i,j\right)=\frac{1}{16}\left[\begin{array}{ccc} 3 & 10 & 3\\ 0 & 0 & 0\\ -3 & -10 & -3 \end{array}\right]*I\left(i,j\right)$$

where $SGM_{x}$ and $SGM_{y}$ denote the horizontal and vertical Scharr gradient magnitudes, respectively. * denotes the convolution operation.

We select the Scharr gradient magnitude as the activity-level measurement index for the visual saliency metric. Consequently, the visual saliency map (VSM) is given as:

(16) $$S\left(i,j\right)=\sqrt{SGM_{x}{\left(i,j\right)}^{2}+SGM_{y}{\left(i,j\right)}^{2}}.$$

**Figure 3:**
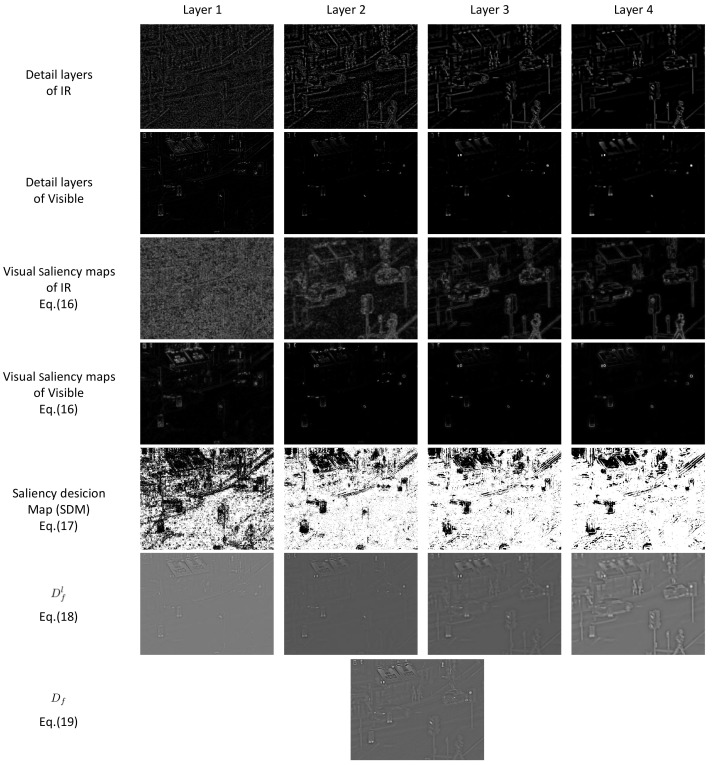
Four-level detail layers fusion process using visual saliency.

In the third and fourth rows of Figure 3, it can be seen that, for detail layers of the IR/Visible images (the first and second rows of Figure 3, respectively), VSM can enhance the detail textures and capture the significant structures of objects from the outline aspect.

The saliency decision map $SDM_{dn}^{l}\left(i,j\right)$ for *N* source images at the *l*-th level is defined as follows:

(17) $$SDM_{dn}^{l}\left(i,j\right)=\left\{\begin{array}{cc} \; & 1,\;if\;S_{dn}^{l}\left(i,j\right)=max\left(S_{d1}^{l}\left(i,j\right),...,S_{dN}^{l}\left(i,j\right)\right)\\ \; & 0,\;otherwise \end{array}\right.$$

where *d* means the detail layer. *n* denotes the *n*-th source image, and *N* is the total number of source images. For instance, $S_{dn}^{l}\left(i,j\right)$ indicates the VSM value of the detail layer for the *n*-th input image at the *l*-th level. The fifth row in Figure 3 shows the saliency decision map (weight map) for each detail layer of the IR image. Obviously, the binary weight values of the saliency decision map for each detail layer of the visible image are complementary to the ones of the IR image.

The combined results of the detail layers (see the sixth row in Figure 3) at the *l*-th level based on the saliency decision map $SDM_{dn}^{l}\left(i,j\right)$ are given by:

(18) $$D_{f}^{l}\left(i,j\right)=\sum_{n=1}^{N}SDM_{dn}^{l}\left(i,j\right)I_{dn}^{l}\left(i,j\right)\;\;n=1,...,N.$$

Finally, the fused detail layer image $D_{f}$ (see the last row in Figure 3) is obtained by:

(19) $$D_{f}=\sum_{l=1}^{L}D_{f}^{l}\left(i,j\right)\;\;l=1,...,L.$$

### 3.4. Reconstruction

By means of combining the fused base layer $B_{f}$ (Equation (13)) and detail layer $D_{f}$ (Equation (19)), the final fused result *F* is reconstructed:

(20) $$F=B_{f}+D_{f}.$$

**Figure 4:**
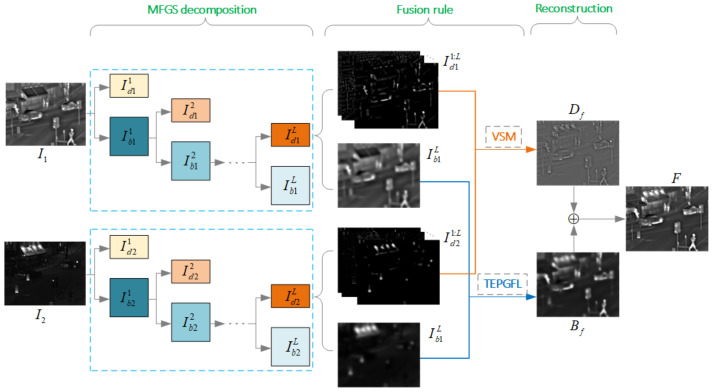
The MFGS fusion framework based on TEPGFL and VSM fusion strategies. Source images are decomposed into a series of detail layers ($I_{dn}^{1:L}$) and a base layer ($I_{bn}^{L}$). Then, with two fusion rules (TEPGFL and VSM), the final fused image *F* is reconstructed by combining the fused detail layer ($D_{f}$) and base layer ($B_{f}$).

Figure 4 schematically illustrates the MFGS fusion framework based on TEPGFL and VSM fusion strategies.

## 4. Experimental Results and Discussion

### 4.1. Experimental Setting

#### 4.1.1. Other Fusion Methods for Comparison

In order to verify the effectiveness and superiority of our MFGS fusion method, a significant amount of experiments are conducted to compare the proposed method with nine state-of-the-art fusion methods including NSCT [33], HyMSD (hybrid multi-scale decomposition with Gaussian and bilateral filters) [23], CSR (convolutional sparse representation) [14], GTF (gradient transfer fusion) [15], VSMWLS (visual saliency map and weighted least square optimization) [24], CNN (convolutional neural networks) [55], DLVGG (deep learning framework using imagenet-vgg-verydeep-19) [40], ResNet (deep learning framework based on ResNet and zero-phase component analysis) [41], and TE (target-enhanced) [51]. The first scheme is a frequently-used and representative MST-based method so far, while the latter six schemes are state-of-the-art methods proposed in recent years.

The experimental setup of our method is completed as follows. The decomposition level is set to 4 referring to [56], which is sufficient to obtain a well-pleasing fusion performance. Although increasing the decomposition level may extract more structural information, it is time consuming. Through numerous experiments, the parameters are set as follows: $\sigma_{c}$ = 0.01, $C_{bf}$ = 0.63. More details of parameter settings for $\sigma_{c}$ and $C_{bf}$ will be provided in Section 4.1.4. For making a fair comparison, the experimental parameters of NSCT are set in the light of [57], and the experimental setup of HyMSD, CSR, GTF, VSMWLS, CNN, DLVGG, ResNet, and TE are set as the original papers, respectively. All the experiments are conducted on a computer equipped with an Intel(R) Core(TM) i5 CPU (2.5 GHz) and 8-GB RAM. The software environment is MATLAB R2018a installed on a Win10 64-bit operating system.

#### 4.1.2. Image Database

A total of 21 per-registered IR and visible image pairs, collected from [41,58], are chosen as the testing data. The TNO image fusion dataset containing multi-spectral nighttime scenarios was established by Dr. Alexander Toet (TNO, Soesterberg, the Netherlands). Interested readers can refer to the REFRENCES sections in the TNO Image Fusion Dataset folders for more details [58]. Furthermore, image registration, as a preprocessing step in image fusion tasks, plays an important role in fusion performance. For more information about the image registration topic, many excellent registration algorithms can be acquired from [59,60]. In addition, a Nato-camp sequence containing 32 IR and visible image pairs is also tested. Throughout this paper, we assume that all source images are perfectly aligned in advance.

#### 4.1.3. Assessment Metrics

Different evaluation metrics (methods) are applied to reflect the fusion image performance in different ways, thus many different assessment methods would be simultaneously employed in the IR and visible image fusion field [1]. In this work, nine commonly-used evaluation metrics are selected for quantitatively and objectively assessing the performances of various fusion methods, including standard deviation (SD), entropy (EN), spatial frequency (SF) [2], tone mapped image quality index (TMQI) [61], visual information fidelity (VIF) [62], sum of the correlations of differences (SCD) [63], average gradient (AG) [64], edge based on similarity measure (Q ${}_{abf}$) [65], and quality metric proposed by Chen and Varshney (Q${}_{cv}$) [66].

SD reflects the brightness differences and contrast of the fused image. EN is usually utilized to measure the information amount of an image. SF is commonly used to measure the clarity and gradient distribution of an image, thereby reflecting the texture and detail of the fused image. TMQI combining the advantages of the naturalness index and the SSIM (structural similarity index measure) index can reflect both the visual naturalness of the fused image and the structural similarity between the fused image and source images. VIF can effectively measure the information fidelity between two source images and the fused image. SCD represents how much information is transferred from the two source images to the fused image. AG is always used to evaluate the image sharpness. Q ${}_{abf}$ quantifies the edge information transferred from source images to the fused image. Q${}_{cv}$ is an image performance metric based on the human vision system (HVS).

For the first eight metrics, the fused result with a large value can achieve a good performance. However, the smaller the Q${}_{cv}$, the better the quality of the fused result.

#### 4.1.4. Parameter Analysis

(i) *Rang parameter $\sigma_{c}$*: For our MFGS fusion method, the fast global smoother is used as the multi-scale decomposition tool to extract the base and detail layers in Section 3.1.2. In this section, we introduce how to choose the rang parameter $\sigma_{c}$ of the fast global smoother. When we test the influence of the parameter $\sigma_{c}$ on the objective metrics, the contrast enhancement coefficient $C_{bf}$ is set to 0.63.

In Section 3.1.2, $\sigma_{c}^{l}$ is the rang parameter at the *l*-th level, and *l* is the multi-scale decomposition level currently. Moreover, $\sigma_{c}^{l}$ is also regarded as the scale control factor. Let $\sigma_{c}^{l+1}=2\sigma_{c}^{l}$ in Equation (6) for extracting the progressively coarser structures. Here, $\sigma_{c}$ denotes the initial value $\sigma_{c}^{1}$.

**Figure 5:**
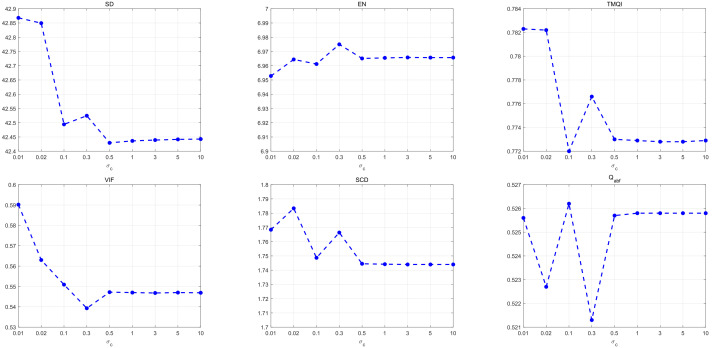
Influence of the parameter $\sigma_{c}$ on the objective metrics. The average values of each metric are obtained by testing the 21 image pairs. From left to right, $\sigma_{c}$ = 0.01, 0.02, 0.1, 0.3, 0.5, 1, 3, 5, 10.

As shown in Figure 5, the average values of each metric are obtained testing on the 21 image pairs. The proposed method achieves the three best values (SD, TMQI, and VIF) when $\sigma_{c}$ is 0.01. Although the peak values of EN, SCD, and $Q_{abf}$ are not obtained at $\sigma_{c}$ = 0.01, the decomposition strategy still achieves a competitive performance when $\sigma_{c}$ is 0.01. All metrics are beginning to stabilize when $\sigma_{c}$ is more than 0.5. Hence, $\sigma_{c}$ = 0.01 is selected as the optimal value to extract the base and detail layers.

(ii) *Contrast enhancement coefficient $C_{bf}$*: In Section 3.2, the key in the final merged base layer, namely $B_{f}=B_{f}=C_{1}B_{f1}\left(i,j\right)+C_{2}B_{f2}\left(i,j\right)=C_{bf}\left(B_{f1}\left(i,j\right)+B_{f2}\left(i,j\right)\right)$ (Equation (13)), lies in the selection of contrast enhancement coefficient $C_{bf}$. Generally, the average of $\left(B_{f1}\left(i,j\right)+B_{f2}\left(i,j\right)\right)$ can achieve good fusion results if $C_{bf}$ is 0.5. Nevertheless, quantitative evaluation using different metrics can reveal more quality information of the fusion results, thereby guiding to tune the parameters of fusion methods. To improve the performance of the fusion results further, we design a contrast enhancement coefficient $C_{bf}$ by conducting sufficient experiments. Obviously, the average scheme ($C_{bf}$ = 0.5) is one of the cases for $C_{bf}$. When we test the influence of the parameter $C_{bf}$ on the objective metrics, the rang parameter $\sigma_{c}$ is set to 0.01.

**Figure 6:**
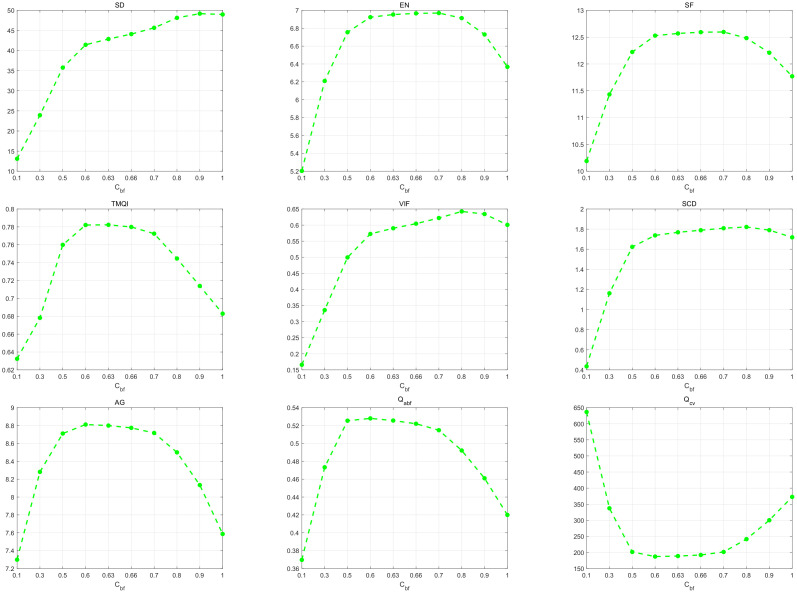
Influence of the parameter $C_{bf}$ on the objective metrics. The average values of each metric are obtained testing on the 21 image pairs. From left to right, $C_{bf}$ = 0.1, 0.3, 0.5, 0.6, 0.63, 0.66, 0.7, 0.8, 0.9, 1.

Figure 6 presents the influence of the parameter $C_{bf}$ on the objective metrics testing on the 21 image pairs. EN, SF, TMQI, SCD, AG, $Q_{abf}$, and $Q_{cv}$ can achieve the best (or competitive) performances when the $C_{bf}$ range is [0.6, 0.66]. The values of SD and VIF at $C_{bf}$ = 0.63 are slightly lower than the peak values of SD ($C_{bf}$ = 0.9) and VIF ($C_{bf}$ = 0.8), respectively. However, if $C_{bf}$ is more than 0.66, especially more than 0.7, the performances of seven other metrics will get worse. Combining the performances of various metrics mentioned above, $C_{bf}$ = 0.63 is chosen as the optimal value.

### 4.2. Quality Performance Comparison on Fusion Rules

In our MFGS fusion method, we propose two different fusion rules (TEPGFL and VSM) to combine the base layers and detail layers, respectively. Typically, the popular “average” (AVG) rule is often used to merge the base layers, and the widely-used “maximum” (MAX) selection or “absolute maximum” (ABSMAX) selection rule is usually employed to merge the detail layers. AVG-MAX and AVG-ABSMAX are two common forms of combination. In addition, the single Gaussian fuzzy logic (SGFL)-based fusion rule is also selected as a competitor to demonstrate the virtue of our TEPGFL-VSM fusion rule. To be fair for comparison, we compare TEPGFL-VSM with AVG-MAX, AVG-ABSMAX, and SGFL-VSM in our MFGS decomposition framework with the same setting.

**Figure 7:**
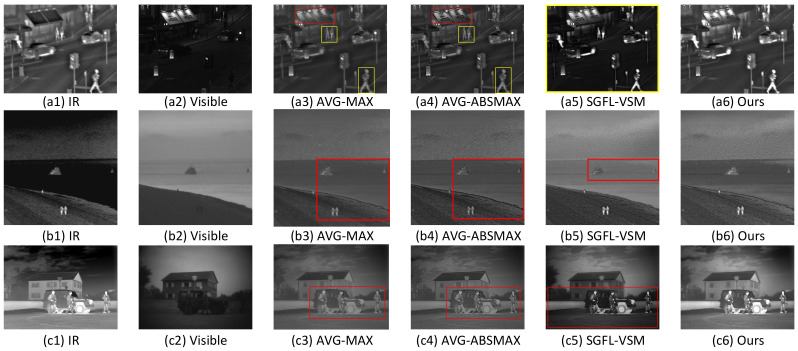
Quality performance comparison on the results of different fusion rules in the MFGS decomposition framework. From top to bottom: ‘Road’, ‘Kayak’, and ‘Soldiers with jeep’.

Figure 7 presents the visual effect comparison of the four types of combination rules tested on ‘Road’, ‘Kayak’, and ‘Soldiers with jeep’. It can be easily seen from Figure 7(a3)–(a5) that there are obvious weaknesses in the results of AVG-MAX, AVG-ABSMAX, and SGFL-VSM. Firstly, for the advertising board ‘NERO’ (red box) and persons (yellow box) in Figure 7(a3,a4), there exist serious brightness distortions, thus making the targets hard to distinguish from the backgrounds. The main causes of these phenomena lie in the fact that using the AVG rule in the base layers can bring about a sharp reduction in contrast. In Figure 7(a5), there has no the above problems. However, the overall background of SGFL-VSM (all enclosed with yellow box) is so dark that it is unnatural and inconsistent with human vision characteristics. Secondly, in Figure 7(a3) (see the red rectangle in Figure 7(a3)), the billboard ‘NERO’ is filled with obscure artifacts and ‘NERO’ is almost indistinguishable. It means that the MAX selection rule is hardly capable of fusing all the detailed information. Furthermore, similar to ‘Road’, ‘Kayak’ and ‘Soldiers with jeep’ suffer from these issues more or less. By contrast, our TEPGFL-VSM scheme can restrain the results from contrast distortion well and achieve nearly artifact-free performance. Due to considering both the target enhancement and salient detail preserving, fused images with our fusion rules are more natural and suitable for human visual perception.

In addition, Table 1 provides the corresponding quantitative evaluation for 3 source image pairs (‘Road’, ‘Kayak’, and ‘Soldiers with jeep’) of various fusion rules. The highest value standing for the best performance is highlighted in bold for each metric except for Q${}_{cv}$. On the contrary, a low value of Q${}_{cv}$ indicates that the fused image has a good performance. From the average values of each metric for 3 source image pairs (‘Road’, ‘Kayak’, and ‘Soldiers with jeep’) of various fusion rules in Table 1, our TEPGFL-VSM fusion scheme outperforms three other fusion rules.

### 4.3. Subjective and Objective Assessments

The subjective evaluation analysis is presented in Section 4.3.1, and the objective assessment analysis is provided in Section 4.3.2.

#### 4.3.1. Subjective Evaluation on the Fused Results

**Figure 8:**
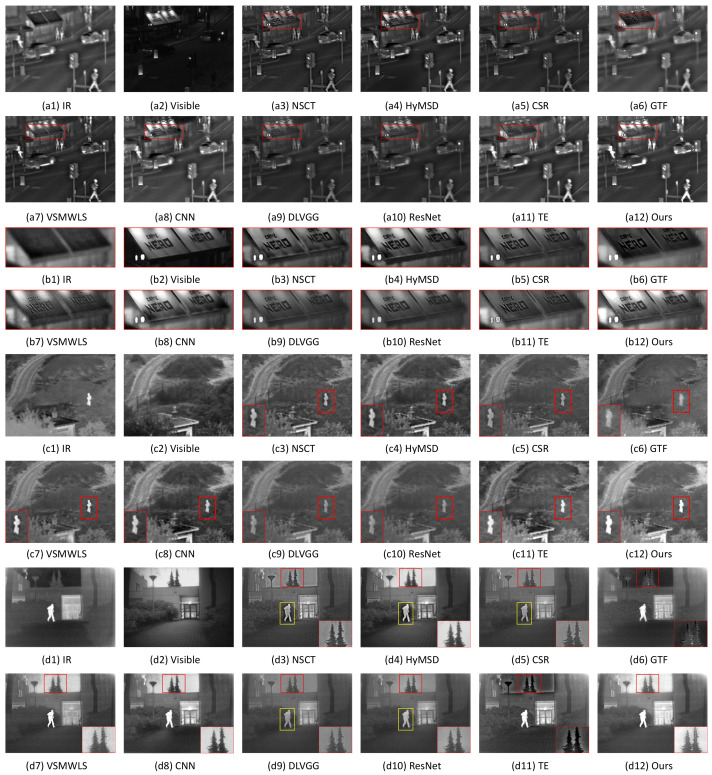
Fusion performance of various methods on the image pairs. From top to bottom: ‘Road ’, close-up view of ‘NERO’, ‘Camp’, and ‘Kaptein’.

The first three groups are ‘Road’, ‘Camp’, and ‘Kaptein’, as shown in Figure 8. Figure 8(a1) represents the IR image ‘Road’ typically containing the significant thermal radiation information of objects, i.e., the persons, cars, and road. However, there has low resolution and insufficient details of the billboard ‘NERO’ and lighting. In contrast, the visible image (Figure 8(a2)) can provide considerable details and high sharpness of the billboard ‘NERO’ and lighting, but the absence of objects information leads to pointlessness of the scene. In Figure 8(a3–a11), all the results are able to fuse the complementary information in both IR and visible images well. However, as shown in Figure 8(a3,a5,a9, and a10), the pedestrians are a little dimmer than the ones in the IR source image and other fusion methods (HyMSD, GTF, VSMWLS, CNN, TE, and Ours). In Figure 8(a3–a11), with the exception of HyMSD and CNN, the billboards ‘NERO’ with brightness distortion are inappropriate for human visual perception as a result of the contrast loss, which is especially serious in GTF, VSMWLS, DLVGG, and TE (see the close-up views in the red boxes in Figure 8b). However, as can be seen from Figure 8(b4,b8), HyMSD and CNN have black stains under two words ‘NERO’, but our result in Figure 8(b12) is able to restrain most black stains. On the contrary, the proposed method achieves better contrast and definition, and also highlights the targets well. It is mainly because our method is capable of suppressing contrast distortion and retaining the sharpness well. For the second case ‘Camp’, in the red boxes of Figure 8c, it is self-evident that there exist black halos around the targets in NSCT and HyMSD, brightness degradation in CSR, GTF, DLVGG, and ResNet, and target blurring in GTF. Nevertheless, VSMWLS, CNN, TE, and our scheme can highlight the target and achieve good visual effects. For ‘Kaptein’ in Figure 8d, GTF, VSMWLS, CNN, ResNet, TE, and Ours acquire relatively satisfying fusion results of the target person. However, in addition to CNN, VSMWLS, and our approach, the other seven schemes suffer from contrast distortions for the sky (see the close-ups in red boxes) and/or person (see the yellow rectangles), thereby being visually unnatural and unpleasing for human vision. For CNN, VSMWLS, and our approach in ‘Camp’ and ‘Kaptein’, it is hard for observers to estimate who is winning or losing only by focusing on subjectively visual effects.

**Figure 9:**
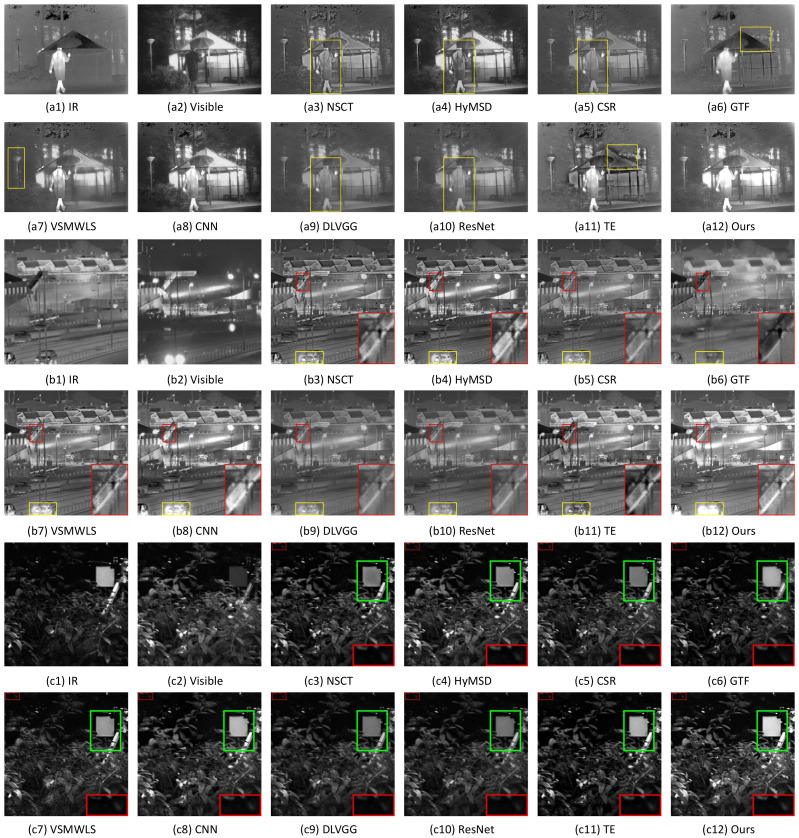
Fusion performance of various methods on the image pairs. From top to bottom: ‘Kaptein1654’, ‘Factory’, and ‘S1’.

In Figure 9(a3–b9), objectively speaking, all fusion methods are able to provide complementary and significant information about the person in the IR image and the igloo in the visible image. But the persons in NSCT, HyMSD, CSR, DLVGG, and ResNet are insufficiently prominent. The street lamps in GTF and VSMWLS are unclear, and the roofs in GTF and TE are slightly dim (see the yellow boxes in Figure 9a, respectively). In regard to ‘Factory’ in Figure 9b, it can be seen from the close-ups of the red rectangles in GTF and TE that black stains fill in the ‘diagonal’ due to introducing too much ambiguous information from the IR image. Furthermore, the lights of the car are hardly found in NSCT, CSR, GTF, DLVGG, ResNet, and TE (see the yellow boxes). Generally speaking, VSMWLS obtains the relatively satisfactory results in both the ‘diagonal’ and the lights of the car. Nevertheless, CNN and our method have more superiorities in such areas as preserving the edge of the ‘diagonal’ and highlighting the salient regions in the IR and visible images (see Figure 9(b8,b12)). Figure 9c shows the performances of various fusion methods in highlighting the targets (see the green boxes) and preserving details (see the close-ups in the red boxes).

**Figure A1:**
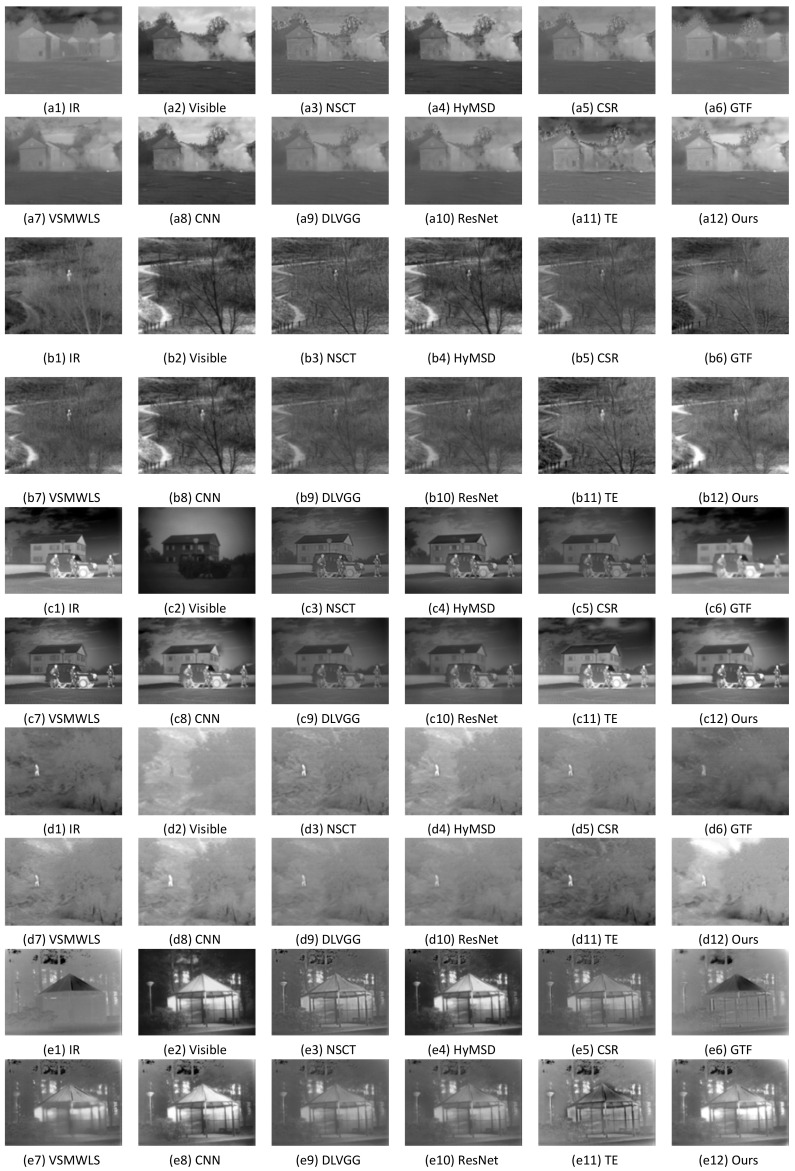
Fusion performances of various methods on the IR and visible image pairs. From top to bottom: ‘Octec’, ‘Sandpath’, ‘Soldiers with jeep’, ‘Trees4906’, and ‘Kaptein19’.

**Figure A2:**
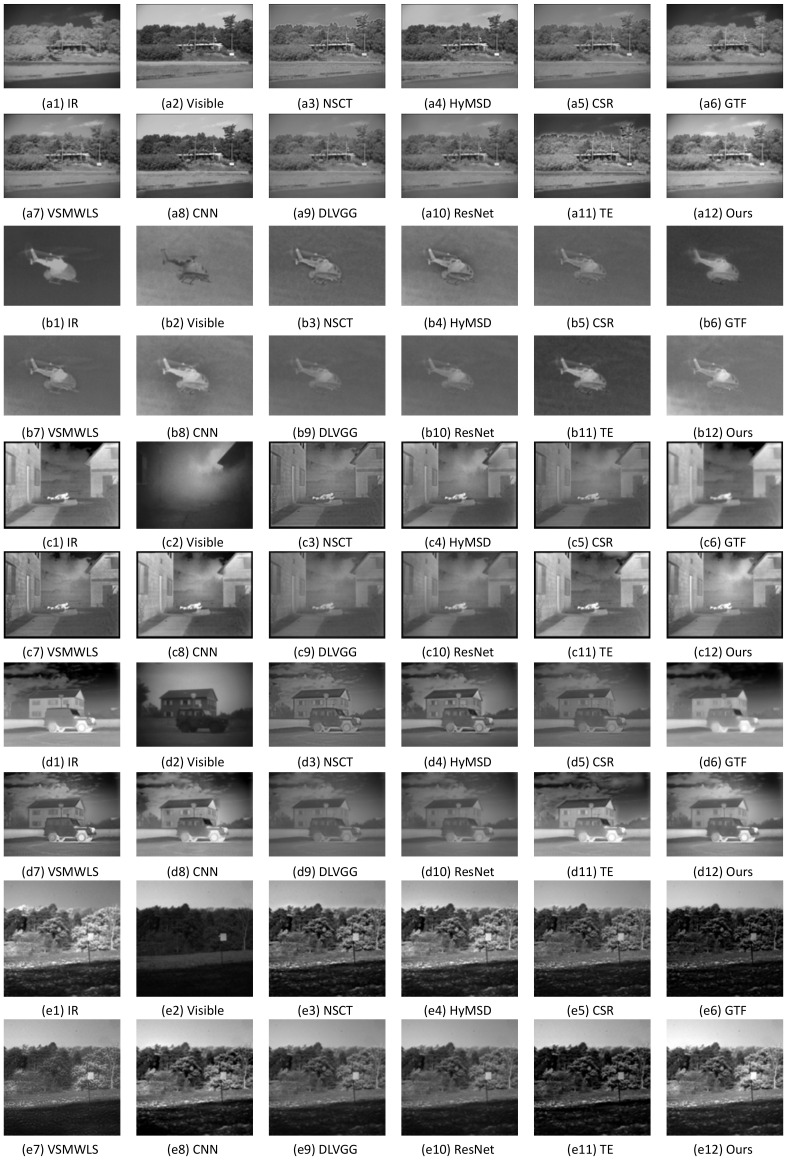
Fusion performances of various methods on the IR and visible image pairs. From top to bottom: ‘Pancake house’, ‘Helicopter’, ‘Soldier behind smoke’, ‘Marne04’, and ‘S2’.

**Figure A3:**
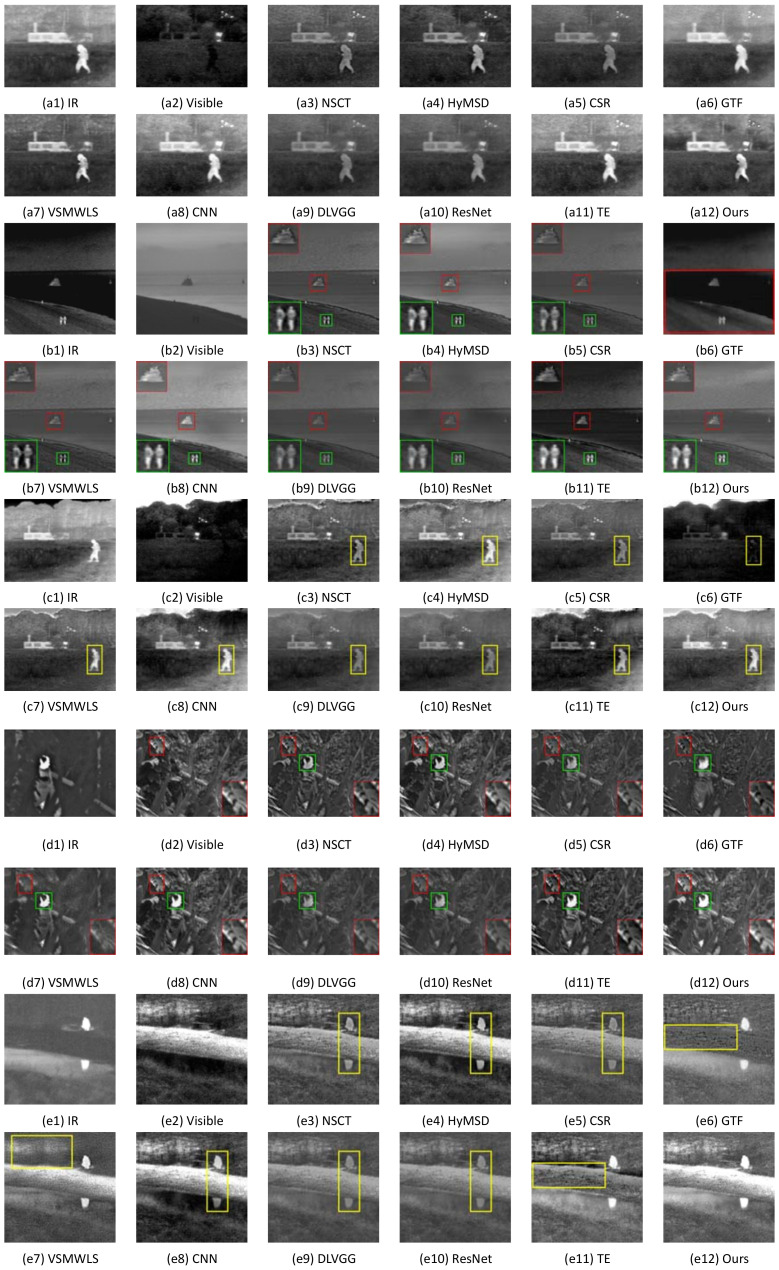
Fusion performances of various methods on the IR and visible image pairs. From top to bottom: ‘Pedestrian’, ‘Kayak’, ‘T1’, ‘T2’, and ‘Bench’.

Furthermore, to fully verify the effectiveness of our fusion algorithm, more experiments of 15 other image pairs are conducted. The fusion performances of various methods on 15 other IR and visible image pairs are illustrated in Figure A1, Figure A2 and Figure A3 in Appendix A. Please refer to Appendix A for more information.

According to the subjective assessment and the detailed analysis mentioned above, now it can be found that our MFGS method is capable of achieving competitive or superior performances compared with several state-of-art methods in qualitative evaluation.

#### 4.3.2. Objective Evaluation on the Fused Results

**Figure 10:**
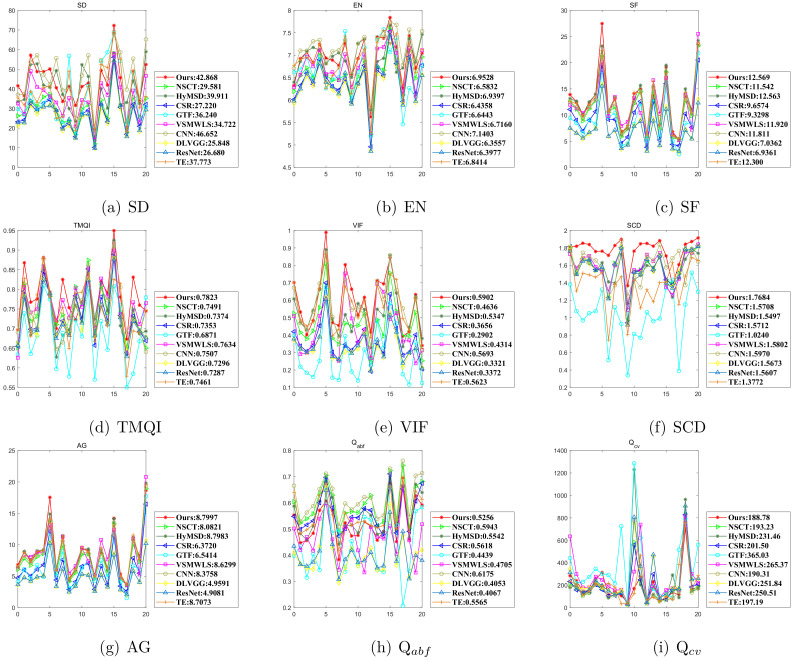
Quantitative comparisons of nine metrics using nine representative methods on 21 IR and visible image pairs. For all methods, the average values are given in the legend. From 0 to 20: ‘Road’, ‘Camp’, ‘Kaptein’, ‘Kaptein1654’, ‘Factory’, ‘S1’, ‘Octec’, ‘Sandpath’, ‘Soldiers with jeep’, ‘Trees4906’, ‘Kaptein19’, ‘Pancake house’, ‘Helicopter’, ‘Soldier behind smoke’, ‘Marne04’, ‘S2’, ‘Pedestrian’, ‘Kayak’, ‘T1’, ‘T2’, and ‘Bench’. (**a**) SD, (**b**) EN, (**c**) SF, (**d**) TMQI, (**e**) VIF, (**f**) SCD, (**g**) AG, (**h**) Q${}_{abf}$, (**i**) Q${}_{cv}$.

Although subjective evaluation can provide a person with intuitive comparisons, it ignores certain imperceptible latent details. Meanwhile objective evaluation using different metrics can reveal more quality information of the fusion results. Each evaluation metric can assess fusion image performance in only one aspect. So when decision makers make a precise and comprehensive decision, all scores should be taken into account. Figure 10 illustrates the objective comparisons using the nine metrics in detail, which are conducted on nine representative methods on the 21 IR and visible image pairs. For all methods, the average values of nine metrics are given in the legend, respectively. As is shown in Figure 10, the proposed method is superior to other fusion approaches in TMQI, VIF, and SCD, while CNN has advantages over other fusion methods in EN and Q${}_{abf}$. For SD, SF, AG, and Q${}_{cv}$, it is hard to distinguish who is the winner because the best values appear alternately among various algorithms.

For the convenience of observation, these average values in the legend in Figure 10 are specially shown in Table 2. The highest value standing for the best performance is highlighted in bold for each metric except for Q${}_{cv}$. The second highest value standing for the second best performance is marked with an underline. It should be noted that a low value of Q${}_{cv}$ indicates that the fused image has a good performance. From Table 2, it can be seen that our method achieves the six best average values in SF, TMQI, VIF, SCD, AG, and Q${}_{cv}$, two second-best metrics in SD and EN, and one intermediate value in Q${}_{abf}$. CNN obtains three best average values in SD, EN, and Q${}_{abf}$ and three second-best metrics in VIF, SCD, and Q${}_{cv}$. HyMSD acquires two second-best metrics in SF and AG. Meanwhile, VSMWLS and NSCT gain the second-best average values in TMQI and Q${}_{abf}$, respectively.

**Figure 11:**
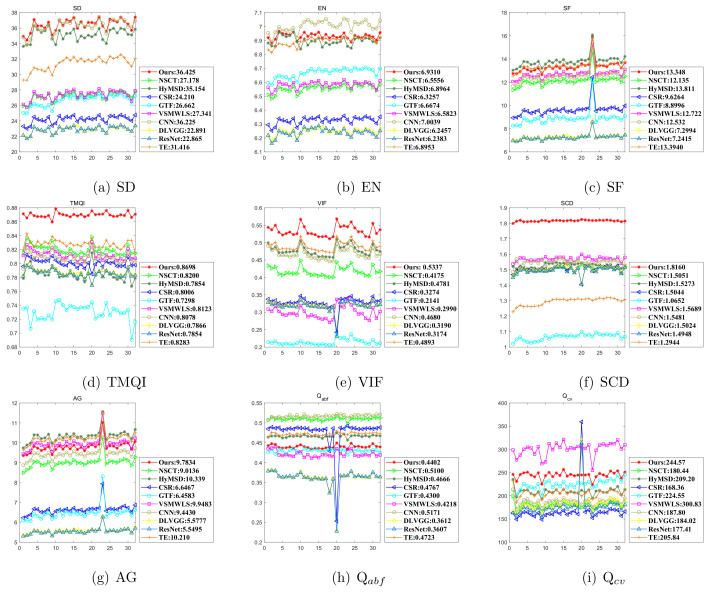
Quantitative comparisons of the nine metrics using nine representative methods on the Nato-camp sequence. For all methods, the average values are shown in the legend. (**a**) SD, (**b**) EN, (**c**) SF, (**d**) TMQI, (**e**) VIF, (**f**) SCD, (**g**) AG, (**h**) Q${}_{abf}$, (**i**) Q ${}_{cv}$.

In addition, to make a further comparison, we present the evaluating results of the Nato-camp sequence, which consists of 32 IR and visible image pairs [58]. As show in Figure 11, the top three places of each metric are: Ours, CNN, and HyMSD in SD; CNN, Ours, and HyMSD in EN; HyMSD, TE, and Ours in SF; Ours, TE, and NSCT in TMQI; Ours, TE, and HyMSD in VIF; Ours, VSMWLS, and CNN in SCD; HyMSD, TE, and VSMWLS in AG; CNN, NSCT, and CSR in Q${}_{abf}$; and CSR, ResNet, and NSCT in Q${}_{cv}$. The findings are arranged in rank order according to the frequency of occurrence, thereby ranking as: Ours (6), HyMSD (5), and CNN, tying with TE (4), etc.

Considering the quantitative analyses of Figure 10 and Figure 11 and Table 2, the objective assessment can support the subjective evaluation well. In comparison with other nine state-of-the-art fusion approaches, extensive comparative experiments demonstrate that our MFGS-based fusion scheme can attain better performance qualitatively and quantitatively.

### 4.4. Experiments on Multiple Images with Different Spectra

Although it is evident that our MFGS-based fusion method obtains a good effect on long wave infrared (LWIR, conventionally called IR before this section in this paper) and visible images in Section 4.3, it is still limited to two imaging modalities. In fact, there are usually multiple (more than two) images needed to synthesize with the purpose of getting a more comprehensive depiction of a scene. Therefore, it is worthy to further discuss the fusion performance of multiple-modality images with different spectra accordingly.

**Figure 12:**
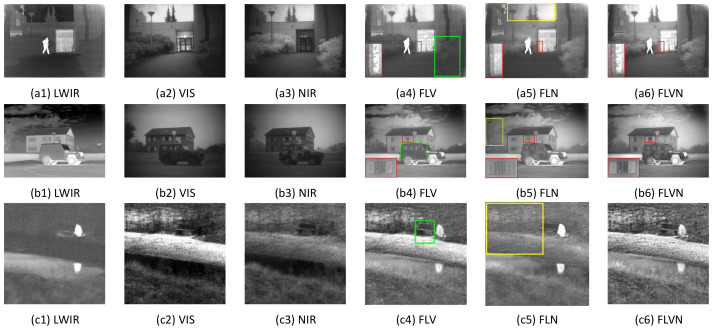
Fusion performance of the proposed fusion method on multiple-modality images with different spectra. LWIR: Long wave infrared image; VIS: Visible image; NIR: Near infrared image; FLV: Fusion image of LWIR and VIS; FLN: Fusion image of LWIR and NIR; FLVN: Fusion image of LWIR, VIS, and NIR. (**a1**) LWIR, (**a2**) VIS, (**a3**) NIR, (**a4**) FLV, (**a5**) FLN, (**a6**) FLVN, (**b1**) LWIR, (**b2**) VIS, (**b3**) NIR, (**b4**) FLV, (**b5**) FLN, (**b6**) FLVN, (**c1**) LWIR, (**c2**) VIS, (**c3**) NIR, (**c4**) FLV, (**c5**) FLN, (**c6**) FLVN.

In this section, for instance, Figure 12 conducts our method on three groups of multi-modality images including long wave infrared (LWIR), visible (VIS), and near infrared (NIR). Here, FLV denotes the fused result of LWIR and VIS; FLN represents the fusion result of LWIR and NIR; and FLVN indicates the fusion result of LWIR, VIS, and NIR. Experimental settings are identical with the ones in Section 4.1. Taking Figure 12a as an example, it shows different fusion results of ‘Kaptein01’. FLV (Figure 12(a4)) cannot display the bright details of the trees (see the green box). Nevertheless, FLN (Figure 12(a5)) cannot exhibit the clear leaf shapes (see the close-up in the red box) and artifact-free sky (see the yellow box). The reason for these imperfections is that FLV and FLN only integrate two source images, thereby lacking enough detailed information congenitally. In contrast, FLVN (Figure 12(a6)) has no these defects mentioned above, and provides a well-pleasing composite image with rich details and high sharpness. Overall, form the fusion results in Figure 12(b4–b6,c4–c6), it can be found that the FLVN images provide abundant details and higher contrast than the FLV and FLN results, thus demonstrating the feasibility and effectiveness of our fusion algorithm for multiple source images.

### 4.5. Computational Efficiency

Besides evaluating the quality, computational efficiency has received increasing attention due to the demands in many practical applications. We report all the time results measured on a PC with a 2.5-GHz CPU and 8 GB of memory. The software environment is MATLAB R2018a installed on a Win10 64-bit operating system. For speeding up our algorithm, we used the C++ programming with a Matlab interface to minimize Equation (1). Table 3 shows the average running time on the Nato-camp sequence images of 360 × 270. To be clear, the training time of two deep learning (DL)-based fusion methods (DLVGG and ResNet) is not taken into account due to using the pre-trained DL network, thereby having low computing costs. In addition to TE, our method outperforms six other methods in computational efficiency. Among them, CSR serving as a machine learning method costs up to 61.282 s because of the considerable learning time using the CSR model. Certainly, using GPU can accelerate CSR, CNN, DLVGG, and ResNet to a large extent, but it will undoubtedly increase the cost, which is also a big restriction for extensive uses of these fusion methods. However, our MFGS-based fusion method takes only 0.4313 s (about 140× faster than CSR). Although TE has less running time than the proposed method, our method still has relatively high computing efficiency. More importantly, the presented method outperforms TE and several state-of-the-art methods in the fusion performance.

## 5. Conclusions and Future Work

In this paper, we successfully applied a multi-scale decomposition and two fusion rules to IR and visible image fusion. The presented fusion method achieved fast and good fusion performances. The main novelties in the presented fusion method are summarized as follows. (i) Multi-scale decomposition based on the fast global smoother is proposed to extract salient information considering both effective edge-preserving and fast detail extraction. The experimental results confirmed that the proposed image decomposition suppresses the halo artifacts well. (ii) Two fusion rules were designed to merge the base and detail layers, respectively. The target-enhanced parallel Gaussian fuzzy logic (TEPGFL)-based fusion rule is proposed to fuse the base layers. The TEPGFL-based fusion rule can overcome the contrast distortions and highlight the important targets and regions. The fusion results are more natural and suitable for human visual perception. The visual saliency map (VSM)-based fusion rule using Scharr gradient is designed to fuse the detail layers. The VSM-based fusion rule can efficiently integrate rich detail textures from multiple source images into the fusion image. Subjective and objective evaluations prove that the fused images have great clarity regarding the details and high visual information fidelity. (iii) The run time comparisons of 10 algorithms demonstrated that the proposed fusion method has high computational efficiency. The high computational efficiency facilitates the practical applications of the IR and visible image fusion. Extensive experiments were conducted on 21 image pairs and a Nato-camp sequence (including 32 image pairs) to verify the effectiveness of the proposed method. Nine state-of-the-art fusion methods were employed for comparison and the results demonstrated that the proposed method can achieve competitive or superior performances according to both the visual effect and objective evaluation. Moreover, we also illustrated the point that the proposed method can be extended to multiple (more than two) source images.

However, the proposed method has a few limitations: (i) In this work, it is assumed that the infrared and visible images are perfectly and strictly registered. In fact, the image preprocessing such as the image denoising and registration in the image fusion algorithms plays a vital role in fusion performance. (ii) The deep learning-based image fusion exhibits a promising trend in the field of image fusion with huge potential for future improvement. (iii) At present the infrared and visible image fusion mainly is applied in video surveillance, agricultural automation, pilot assistance, remote sensing, and biometric identification, etc. The feasibility of infrared and visible image fusion for other practical applications such as the damage detection and identification in structures is rarely considered, which will be studied in our future works. Therefore, there are some works to be worth investigating in the future. As indicated by the first limitation, image preprocessing, such as image registration, can be explored to further improve the performances of fusion methods. From the second limitation, we can develop novel deep neural networks and improve the computational efficiency of the deep learning-based fusion method with a parallel computing unit. Finally, we will also devote to investigating the potential of the infrared and visible image fusion technique to other practical applications.

## Figures and Tables

**Table 1:** Objective assessments. The metric value marked in bold indicates that the fusion rule achieves the best performance on the current image pair. “Average” means the average values of each metric for 3 source image pairs (‘Road’, ‘Kayak’, and ‘Soldiers with jeep’) of various fusion rules.

Images	Rules	SD	EN	SF	TMQI	VIF	SCD	AG	Q${}_{abf}$	Q${}_{cv}$
Road	AVG-MAX	24.413	6.0355	8.5729	0.6659	0.4003	**1.8342**	4.0542	0.4199	378.05
	AVG-ABSMAX	24.445	6.1007	11.907	0.6666	0.4715	1.8230	5.9309	0.5438	**228.23**
	SGFL-VSM	38.103	5.8504	**14.140**	0.6290	0.5068	1.7169	6.6651	0.4740	323.80
	TEPGFL-VSM	**41.557**	**6.3234**	13.905	**0.6966**	**0.7018**	1.8108	**6.7974**	**0.5538**	283.29
Kayak	AVG-MAX	16.252	5.9252	3.5059	0.6383	0.2179	1.5516	2.1879	0.4537	170.13
	AVG-ABSMAX	17.286	6.0503	5.1000	0.6568	0.3488	1.5228	3.5362	**0.7037**	171.35
	SGFL-VSM	**32.229**	**6.4433**	5.1708	0.6337	0.2105	1.4577	**3.5744**	0.6590	**94.326**
	TEPGFL-VSM	22.968	6.2697	**5.2529**	**0.6729**	**0.3935**	**1.6082**	3.5453	0.6958	115.43
Soldiers	AVG-MAX	21.974	6.4668	3.9577	0.6848	0.2962	1.8185	2.8266	0.3782	151.94
with jeep	AVG-ABSMAX	22.460	6.5066	5.3945	0.6979	0.4046	1.8131	4.1455	0.4951	148.13
	SGFL-VSM	34.533	6.9558	6.1089	0.6849	0.4555	1.6023	4.2572	0.4600	330.36
	TEPGFL-VSM	**41.343**	**7.2718**	**6.8693**	**0.7534**	**0.8037**	**1.8985**	**4.6329**	**0.5237**	**105.73**
Average	AVG-MAX	20.880	6.1425	5.3455	0.6630	0.3048	1.7348	3.0229	0.4173	233.37
	AVG-ABSMAX	21.397	6.2192	7.4673	0.6738	0.4083	1.7196	4.5375	0.5809	182.57
	SGFL-VSM	34.955	6.4165	8.4731	0.6492	0.3909	1.5923	4.8322	0.5310	249.50
	TEPGFL-VSM	**35.290**	**6.6216**	**8.6756**	**0.7076**	**0.6330**	**1.7725**	**4.9919**	**0.5911**	**168.15**

**Table 2:** Average values of each metric for 21 source image pairs of various fusion methods. The metric value marked in bold indicates the best performance, and the metric value marked with an underline denotes the second best performance.

	SD	EN	SF	TMQI	VIF	SCD	AG	Q${}_{abf}$	Q${}_{cv}$
NSCT	29.581	6.5832	11.542	0.7491	0.4636	1.5708	8.0821	0.5943	193.23
HyMSD	39.911	6.9397	12.563	0.7374	0.5347	1.5497	8.7983	0.5542	231.46
CSR	27.220	6.4358	9.6574	0.7353	0.3656	1.5712	6.3720	0.5618	201.50
GTF	36.240	6.6443	9.3298	0.6871	0.2902	1.0240	6.5414	0.4439	365.03
VSMWLS	34.722	6.7160	11.920	0.7634	0.4314	1.5802	8.6299	0.4705	265.37
CNN	**46.652**	**7.1403**	11.811	0.7507	0.5693	1.5970	8.3758	**0.6175**	190.31
DLVGG	25.848	6.3557	7.0362	0.7296	0.3321	1.5673	4.9591	0.4053	251.84
ResNet	26.680	6.3977	6.9361	0.7287	0.3372	1.5607	4.9081	0.4067	250.51
TE	37.773	6.8414	12.300	0.7461	0.5623	1.3772	8.7073	0.5565	197.19
Ours	42.868	6.9528	**12.569**	**0.7823**	**0.5902**	**1.7684**	**8.7997**	0.5256	**188.78**

**Table 3:** Average running time of various methods on the Nato-Camp sequence images of 360 × 270 (Unit: second).

	NSCT	HyMSD	CSR	GTF	VSMWLS	CNN	DLVGG	ResNet	TE	Ours
Time	1.8631	1.3315	61.282	1.1098	0.8926	38.538	3.0282	1.8516	0.0797	0.4313

## Data Availability

Not applicable.

## Appendix A

The fusion performances of various methods on 15 other IR and visible image pairs are illustrated in Figure A1, Figure A2and Figure A3. For the last four rows (‘Kayak’, ‘T1’, ‘T2’, and ‘Bench’) in Figure A3, we compare our method with other fusion approaches locally and in detail in a rectangle manner. Here, taking ‘Kayak’ as an example, NSCT has black halos between the two persons and around the boat, and HyMSD and VSMWLS encounter black halos between the two persons as well. Although GTF has the power to highlight the targets, the background of the sea is too dark to distinguish the sea surface from the coasts. The main targets seem to be dim in CSR, DLVGG, and ResNet. Meanwhile, CNN, TE, and our result overcome these issues.
